# Architecture of crossed-lamellar bivalve shells: the southern giant clam (*Tridacna derasa*, Röding, 1798)

**DOI:** 10.1098/rsos.170622

**Published:** 2017-09-06

**Authors:** O. B. A. Agbaje, R. Wirth, L. F. G. Morales, K. Shirai, M. Kosnik, T. Watanabe, D. E. Jacob

**Affiliations:** 1 Department of Earth and Planetary Sciences, Macquarie University, Sydney, NSW 2109, Australia; 2 Helmholtz-Centre Potsdam, German Research Centre for Geosciences GFZ, 14473 Potsdam, Germany; 3 Scientific Center for Optical and Electron Microscopy (ScopeM), ETH Zürich, Auguste-Piccard-Hof 1, HPT D9, 8093 Zürich, Switzerland; 4 International Coastal Research Center, Atmosphere and Ocean Research Institute, The University of Tokyo, 5-1-5, Kashiwanoha, Kashiwa-shi, Chiba 277-8564, Japan; 5 Department of Biological Sciences, Macquarie University, Sydney, NSW 2109, Australia; 6 Department of Natural History Sciences, Hokkaido University, N10W8, Kita-ku, Sapporo 060-0810, Japan

**Keywords:** Bivalvia, Tridacnidae, transmission electron microscopy, electron backscatter diffraction, aragonite, Young's modulus

## Abstract

*Tridacna derasa* shells show a crossed lamellar microstructure consisting of three hierarchical lamellar structural orders. The mineral part is intimately intergrown with 0.9 wt% organics, namely polysaccharides, glycosylated and unglycosylated proteins and lipids, identified by Fourier transform infrared spectrometry. Transmission electron microscopy shows nanometre-sized grains with irregular grain boundaries and abundant voids. Twinning is observed across all spatial scales and results in a spread of the crystal orientation angles. Electron backscatter diffraction analysis shows a strong fibre texture with the [001] axes of aragonite aligned radially to the shell surface. The aragonitic [100] and [010] axes are oriented randomly around [001]. The random orientation of anisotropic crystallographic directions in this plane reduces anisotropy of the Young's modulus and adds to the optimization of mechanical properties of bivalve shells.

## 1. Introduction

Bivalve shells are complex biocomposites consisting of calcium carbonate intimately intergrown at the nanoscale with organic macromolecules [1,2]. This composite nature creates enhanced material properties, for example high mechanical strength [3] and fracture toughness [4,5], which optimize shell stability and protective function for the organism [6]. Much recent research has focused on the nacreous shell structure in molluscs, while other shell structures in this phylum, such as the most widespread crossed lamellar structure [7], are yet to receive comparable attention. Here, we present one of the first in-depth characterizations of both the inorganic and the organic parts in *Tridacna derasa* (southern giant clam) shells. Furthermore, we combine here electron backscatter diffraction (EBSD) with transmission electron microscopy (TEM) analysis to identify some of the multi-scale strategies for the optimization of mechanical properties across all structural hierarchies in the shell.

### 1.1. Structure and micro-texture of *Tridacna derasa* shells

**Figure 1.**
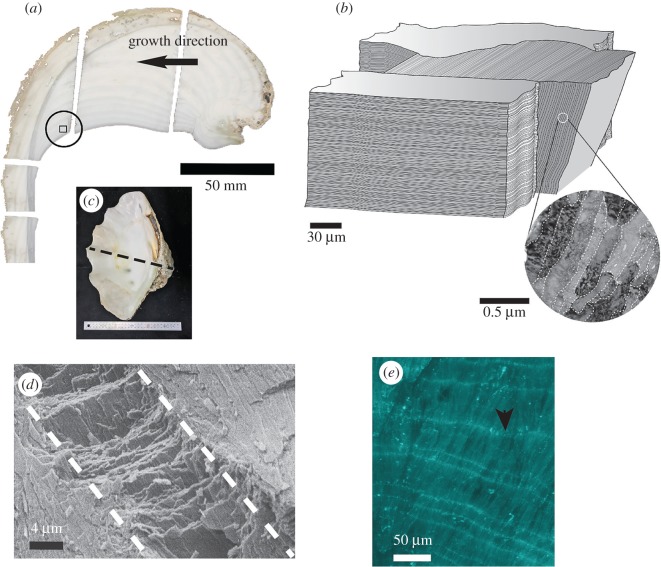
(*a*) Sectioned shell of *T. derasa* showing the location of the EBSD map (circled box). The outer layer, extending approximately a further 10 cm was cut off at the level of the inner shell layer. (*b*) Schematic sketch of the hierarchical lamellar shell structure. An enlargement showing third-order grains is taken from figure 6*c* with grain boundaries outlined by dashed lines. (*c*) Complete valve of the shell with the dashed line indicating where the shell was sectioned. (*d*) SEM image of a fractured surface of the shell shows the first-order lamellae (dashed lines) consisting of second-order laths. (*e*) Organic daily growth lines stained with Calcofluor White (arrow) transect the aragonitic lamellae.

*Tridacna derasa* shells are entirely aragonitic and consist of an approximately 10 mm thick massive outer layer and a slightly translucent inner layer with visible growth increments (figure 1*a,c*). The shell comprises crossed lamellar shell structure, which is the most common structure of mollusc shells and has been described in detail by a number of authors (e.g. [7–13]). With very few exceptions, shells with this structure are aragonitic rather than calcitic [14]. Crossed lamellar shells from different species vary in structural arrangement but bear a basic architectural similarity [15]: aragonite grains are arranged in hierarchically organized lamellae (figure 1*b,d*) with alternating orientations at an angle of approximately 70–90° depending on the species [7,12]. Three or four hierarchical orders can be identified and growth twinning of the aragonite crystals is very common (e.g. [12,16]).

The first structural hierarchy in *T. derasa* shells comprises a series of undulating aragonite bands 20–50 µm thick (first-order lamellae, figure 1*b*) consisting of nanometre-thin stacked second-order lamellae. These sheets of stacked lamellae are oriented at an angle of approximately 70° to each other and are perpendicular to the shell surface. Each sheet of second-order lamellae, in turn, consists of nanometre-sized parallel aragonite laths, which form the third order of hierarchy (figure 1*b*).

*Tridacna* species are known to form daily growth lines [17,18] with growth increments around 15 µm in width. Growth lines in molluscs usually have increased organic content compared with the increments between a set of lines and can be visualized using histochemical staining methods (figure 1*e*).

## 2. Material and methods

The southern giant clam, *T. derasa* (Röding 1798) (Mollusca: Bivalvia), is the second largest species in the family Tridacnidae, reaching shell lengths of up to 520 mm [19]. Tridacnidae occur naturally in the tropical and subtropical waters of the Indo-Pacific and host photosymbiotic algae in their tissues [20]. Shells of *T. derasa*, cultured on Ishigaki Island, Okinawa, Japan (for environmental details see [18]), were used for structural analyses of the inorganic part of the shells, while organic matrix analysis was performed using a recently alive shell from One Tree Island, Queensland, Australia*.*

### 2.1. Scanning electron microscopy and electron backscattered diffraction

Broken pieces of shell were imaged with a Leo Gemini 1530 field-emission secondary electron microscopy (SEM) instrument (Carl Zeiss, Germany) at the Max-Planck Institute for Chemistry, Germany. Samples were mounted on aluminium stubs using a conductive carbon tape. All samples were studied uncoated with an accelerating voltage of 5 kV, a sample current of 2 pA and a vacuum pressure of 5 × 10^−6^ mbar.

Crystallographic preferred orientations (CPO) in the shell were determined by automated indexation of EBSD in a scanning electron microscope [21,22] using an EDAX TSL Digiview 3 EBSD camera and an OIM DC 5.0 detector. The sample was polished using standard methods with diamond pastes of different grain sizes down to 0.25 µm and a final step of chemical–physical polishing using a neoprene polishing cloth and an alkaline solution of colloidal silica for 1 h. The EBSD measurements were conducted in an area of the shell along the axis of maximum growth (figure 1*a*). Analyses were carried out under low vacuum (10 Pa of H_2_O) using the following parameters for SEM: 15 kV accelerating voltage; 8 nA beam current; 12 mm working distance; step size of 1 µm and 70° sample tilt. At these conditions, the electron beam size is 4 nm and approximately 90% of all diffraction patterns could be indexed.

Post-acquisition treatment included the standardization of the confidence index (CI) of different points and CI correlation between neighbouring points. ‘Grain’ dilation was carried out in three steps considering the grain tolerance angle of 10° and a minimum grain size of 10 pixels. Grain sizes as observed by TEM are usually in the nanometre range in biominerals (e.g. figures 4–6), hence the chosen ‘grain’ size cut-off for EBSD defines domains of several crystallographically well-aligned nanograins, rather than individual aragonite grains. These domains have misorientation angles less than 10° and are here termed ‘first-order domains’.

**Figure 2.**
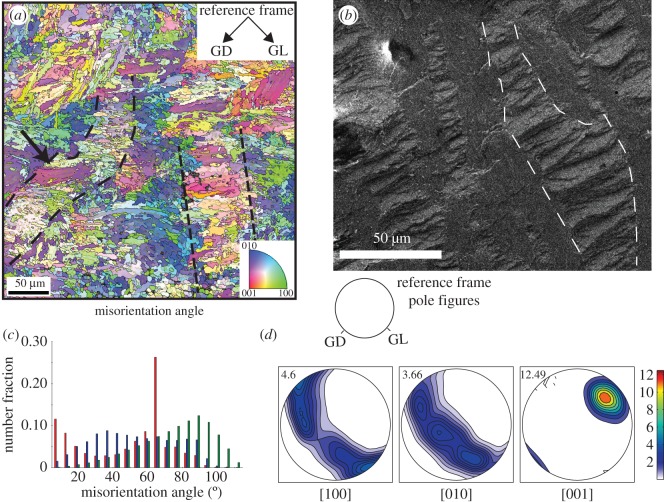
(*a*) Crystallographic orientation map coloured according to the aragonite inverse pole figure colour code (inset). The reference frame (upper right) points out the overall growth direction of the shell (GD) and the direction of the growth lines (GL). For a red domain in the map, the [001] *c*-axis is pointing to the reader; if green, it is the *a*-axis [100]; and if blue, it is the *b*-axis [010]. Dashed lines outline some first-order lamellae. First-order domains are separated by low-angle boundaries within the domains (grey lines) and by high-angle boundaries between different co-oriented areas (black lines). The arrow points to the co-oriented area detailed in figure 3. (*b*) SEM image (fractured shell surface) of a similar area, first-order lamella outlined with dashed lines. (*c*) Histogram of misorientation angles for neighbour (red bars) and non-neighbour (blue bars) grains. Note the predominance of low-angle grain boundaries (misorientations less than 15°) for neighbouring crystals, which is not observed between non-neighbour grains. Distributions of both neighbours and non-neighbour grains are distinctly different from the expected misorientation angles for random grain boundaries (green bars). (*d*) Pole figures of the [100], [010] and [001] axes of aragonite (lower hemisphere of the stereographic projection), showing a strong crystallographic preferred orientation of the [001] axes around the GD, with the [100] and [010] axes forming a single broad girdle parallel to the plane that contains the direction of growth lines (GL, see the inset in (*a*) for the external reference frame). The maximum density of the [001] axes is 12.49 times uniform (value in upper left corner, compare to scale).

The pseudohexagonal symmetry effect on aragonite caused by a rotation of 60° around [001] was also corrected. Data with CI > 0.1 are plotted in pole figures (figure 2*d* and figure 9*b*), which are stereograms with axes defined by an external reference frame using the shell length growth direction (GD), the direction of the growth lines (GL) and the axis normal to these features. Accumulation of points around a specific direction in the pole figures (pole maxima) shows a degree of texture in the polycrystalline material, quantified according to the colour scales in the figures. The rotations of the crystallographic dataset and plots of pole figures were carried out using the MTEX toolbox for Matlab [23,24].

### 2.2. Transmission electron microscopy

TEM foils approximately 10 by 15 µm in length and 0.15–0.20 µm thickness were cut from the polished section of the sample using an FEI FIB200 focused ion beam device (FIB) following procedures given in Wirth [25]. Six foils were cut from the inner and outer layers of the shell, either parallel or perpendicular to the growth lines. Samples were placed on a carbon-coated Cu grid without further carbon coating (*ex situ* lift out method). The FIB milling method involves sputtering the material surrounding the platinum-protected target area with gallium ions. This process can heat the target area, and drive amorphization through Ga implantation in the surface of the material [26]. Sample heating is proportional to the beam current, and the extent of amorphization is proportional to the beam energy; both depend on the angle of beam incidence during milling [27]. To avoid heating of the sample, we used 30 keV with a beam current of 11 pA and an angle of incidence of 1.2°. At these conditions beam heating during FIB milling is less than 10 K [28] and sample amorphization is minimal*.* As the foils are thicker than 100 nm, the major part of the foil is thus not affected by ion implantation. If amorphization were a significant problem in the foils, Debye–Sherrer diffraction rings would be present in all collected diffraction patterns, but these features were not observed.

TEM imaging and analysis were undertaken with a FEI Tecnai™ G2 F20 X-Twin transmission electron microscope with a field emission gun source, operating at 200 kV acceleration voltage. A Gatan Tridiem™ filter allowed energy-filtered imaging, applying a 20 eV window to the zero-loss peak for all bright-field images in this study. Images were taken either in scanning TEM (STEM) mode or in high-angle annular dark field (HAADF) mode with a 330 mm camera length. At these conditions imaging is possible with *z*-contrast, diffraction, thickness and density contrast.

Great care was taken to minimize radiation damage to the material during TEM analysis. This involved low-dose analysis and a visual monitoring protocol developed for biominerals [29,30]. Foils were analysed in STEM mode, rapidly scanning using a small spot size and assigning the beam to areas outside the sample to avoid electron irradiation damage. At the start of the analytical session for each FIB foil, a rapid overview picture was taken using a defocused beam and this was repeated after STEM scanning and high-resolution electron microscopy analysis and at the end of each analytical session to scan for beam damage. All high-resolution TEM analyses were carried out at the end of the analytical session for each foil, using exposure times of 0.2 s and a spot size of 5. Using this protocol, irradiation damage was only observed on a few occasions, manifested either as holes from the electron beam or as localized small amorphized areas where a STEM scan had been carried out. Analyses and images from these areas were discarded from the dataset.

## 3. Organic matrix characterization

### 3.1. Thermogravimetry

The total amount of organic shell matrix was determined by thermogravimetric analysis (TGA) with a TGA 2050 thermogravimetric analyser (TA Instruments, USA) at the Department of Chemistry and Biomolecular Sciences, Macquarie University. Approximately 30 mg of powdered and sieved sample from the inner part of the shell (250 µm mesh size) was measured in a temperature interval from 30°C up to 1000°C at 10°C min^−1^ steps under a nitrogen atmosphere.

### 3.2. Extraction methods

To characterize the organic matrix, the shell was decalcified in 6 N HCl after cutting and removing its outermost part, followed by a cleaning step that involved immersing the shell in 30% H_2_O_2_ (Merck KGaA, Darmstadt, Germany) and rinsing with Milli-Q water. The solution was stored at 4°C after decalcification. The supernatant was extracted twice, first with dichloromethane and then with butanol (BuOH), both fractions were combined and reduced to dryness before storage at −35°C.

The lipid–lipoprotein fraction was extracted with methanol/dichloromethane (2/1) at room temperature for 40 h, ultrasonicated and dried in a nitrogen atmosphere.

An aliquot of the total organic matrix extract was taken up in dimethylacetamide containing 5% lithium chloride [31], centrifuged and filtered. The specific optical activity of the filtrate was analysed with a JASCO P-1010 polarimeter (JASCO, Tsukuba, Japan) at the Department of Chemistry and Biomolecular Sciences, Macquarie University. A cell line of sodium D at 589 nm was used as the filter at room temperature of 21 ± 1°C.

### 3.3. Infrared spectroscopy

Fourier transform infrared (FTIR) spectra of different extracted and dried organic matrix fractions were measured with a Thermo Nicolet iS10 FTIR spectrometer (Nicolet, MA, USA) equipped with attenuated total reflection along with a smart performer assessor at the Department of Chemistry and Biomolecular Sciences, Macquarie University. Spectra were acquired between 4000 and 500 cm^−1^ with a resolution of 8 cm^−1^ and 64 accumulations. Backgrounds were recorded at the beginning of the analytical session and approximately every half hour.

## 4. Results

### 4.1. Electron backscattered diffraction analysis

EBSD was carried out on a polished area of the inner layer of the shell situated between, but not overlapping with, two organic-rich annual growth lines (approximate location outlined in figure 1*a*). Figure 2*a* shows a crystal orientation map for this area. The map is coloured according to the orthorhombic inverse pole figure colour scheme for aragonite (inset), assuming the reader's perspective. Areas in red have their crystallographic [001] axis pointing towards the reader, green denotes the [100] axis and blue the [010] axis. Intermediate colours are crystallographic orientations intermediate between these three extremes. Adjacent areas with a misorientation angle greater than 10° define a boundary outlined in grey in figure 2*a*. These boundaries, however, do not delineate single grains, but rather domains of co-oriented smaller aragonite crystals with grain sizes beyond the spatial resolution of the EBSD method. They are termed here ‘first-order domains’ (see Material and methods).

Two first-order lamellae are outlined with dashed lines in figure 2*a*, and a similar area in the shell is shown in figure 2*b*, where first-order lamellae (dashed lines) consisting of second-order lamellae are arranged in alternating orientation. Twinning is commonly observed at this level of hierarchy in the shell and amounts to approximately 26% of the ‘grain’ (i.e. first-order domain) boundaries (figure 2*c*). This value is derived from analysis of the boundaries between first-order domains with a misorientation angle of 60° in the CPO map (figure 2*a*), which is close to the angle of twinning on {110} in aragonite. It should be noted that the estimated value of 26% is a lower limit for the total amount of twin boundaries, because its precision is determined by the measurement conditions and the spatial resolution of the EBSD analysis. Nevertheless, even this rough estimate shows that aragonite twinning is very common in the shell across all structural hierarchies.

The CPO of the shell shows a strong alignment of [001] axes parallel to the growth direction of the shell (GD) as seen in the pole figures (figure 2*d*), with a maximum of about 12× the uniform distribution. The [100] and [010] axes are distributed at random along continuous and broad single girdles normal to GD, with a weak tendency for [100] to be parallel with the GL direction. Hence, the aragonite crystals are strongly co-aligned in the [001] axial direction but random in radial direction (i.e. concerning their [100] and [010] axes). These are the typical characteristics of a fibre texture.

Figure 2*a* shows that first-order domains forming several second-order lamellae within a given first-order lamella are co-oriented, forming areas of uniform or similar colour (arrow in figure 2*a*). These larger areas with small internal misorientation angles are generally around 50 µm in size and are separated from each other by high-angle first-order domain boundaries (misorientation angle greater than 15°, black lines).

**Figure 3.**
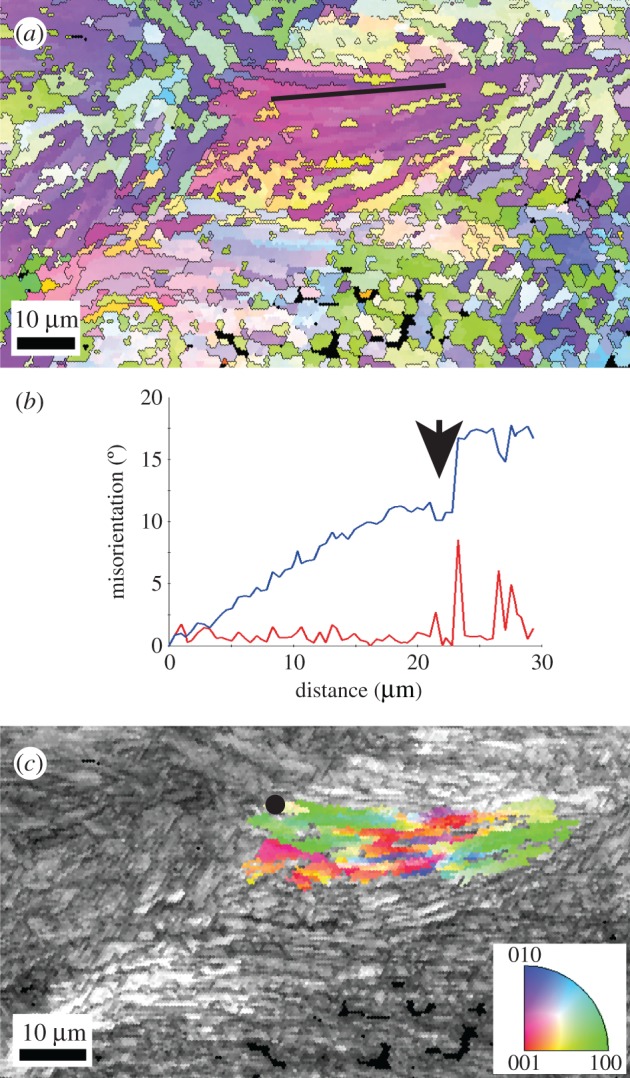
Region of interest marked with a black arrow in figure 2*a*. (*a*) Crystallographic orientation map coloured according to the aragonite inverse pole figure colour code (inset in (*c*)) and detailed in figure 2. (*b*) Misorientation profile (black line in (*a*)) showing change in crystal orientation with a misorientation angle of approximately 15° across approximately 30 µm. Red curve = point to point values, blue curve = point to origin values. Arrow indicates a subdomain boundary. (*c*) Image quality map where different levels of grey indicate the EBSD pattern quality—clear, high quality patterns in light grey, lowest quality patterns in dark grey. In colour, the ‘grain’ reference orientation deviation axes, showing the deviation of crystallographic orientation relative to the black point in the map (colour reference as inset).

Analysis of one such large area (figure 3*a*, marked with an arrow in figure 2*a*) shows misorientation angles of approximately 15° across the entire area of approximately 50 µm (figure 3*b*). Notably, the misorientation axes within this first-order domain vary considerably (figure 3*c*), i.e. they are parallel to different crystal directions that can diverge by as much as 90°. This shows that individual aragonite particles (or groups of particles) within this large area are misoriented to different degrees along individually differing misorientation axes, confirming the particulate nature of the area below the spatial resolution of the EBSD method.

### 4.2. Transmission electron microscopy

**Figure 4.**
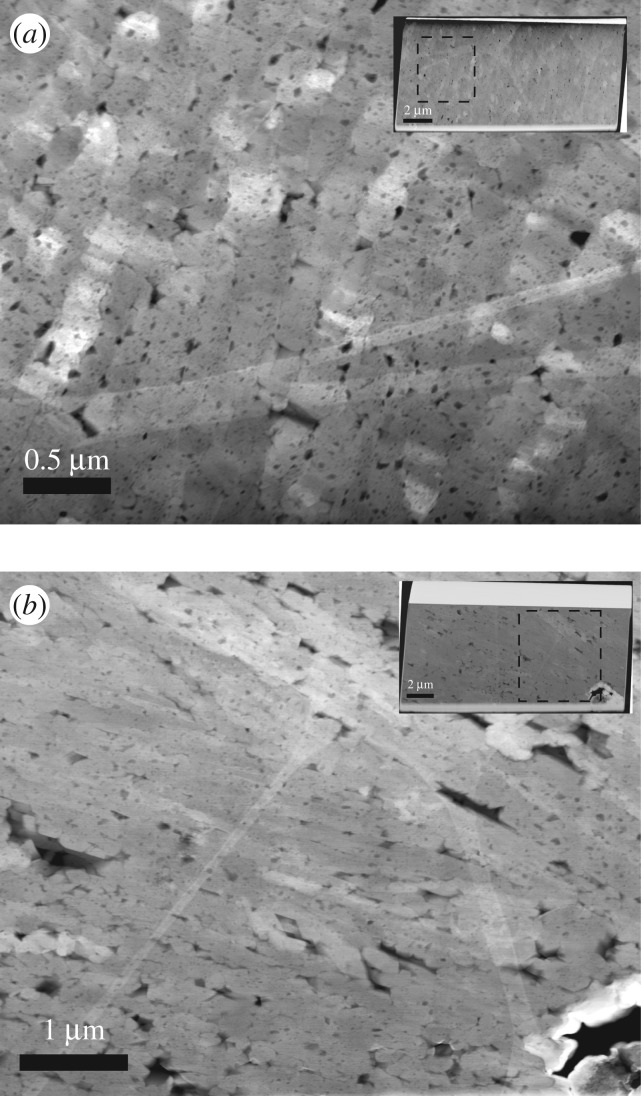
HAADF images of TEM foils cut perpendicular (*a*) and parallel (*b*) to the outer surface of the shell in the outer shell layer (figure 1*a*). Insets show the entire TEM foils with black squares marking the locations of the areas enlarged in (*a*) and (*b*). Note the small aragonite laths cut perpendicular (*a*) and parallel (*b*) to their longest axes and the numerous multi-scale voids.

**Figure 5.**
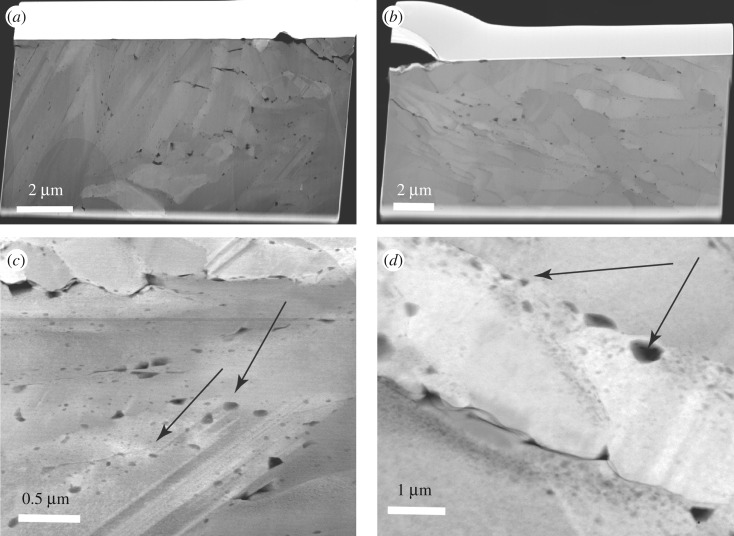
HAADF images of foils cut by FIB milling parallel (*a,c*) and perpendicular (*b,d*) to the outer surface of the shell in the inner shell layer (figure 1*a*). The lath-shaped aragonite grains are cut perpendicular (*a*) and parallel (*b*) to their longest axes. Note the voids along lath boundaries and within the laths (*c,d*). Polycyclic twinning is common (*c*). The different grey shades of the aragonite crystals are differences in diffraction contrast due to different crystal orientations.

**Figure 6.**
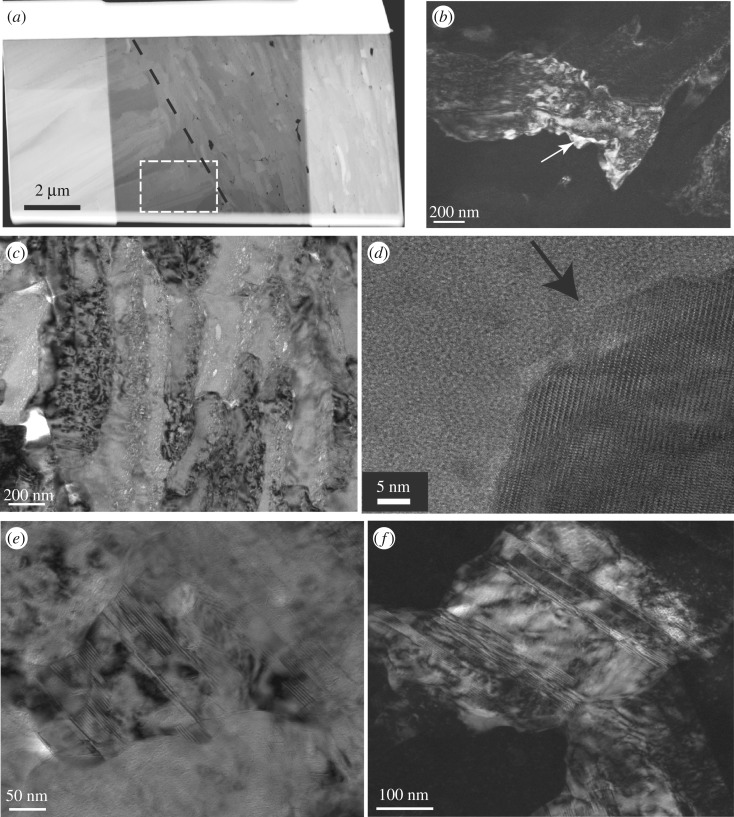
(*a*) TEM foil cut perpendicular to the growth lines in the shell and across an interface (black dashed line) between two first-order lamellae containing second order lamellae of different orientation (foil thinned in the central part, HAADF image, inner shell layer). (*b*) Dark-field image of an aragonite grain outlined with the white dashed box in (*a*). Note the irregular shape and ‘speckled’ diffraction contrast. (*c*) Bright-field image showing numerous inter- and intragranular voids similar to those shown by STEM imaging in figure 5*c,d*. High resolution TEM image of the tip (*d*) of a third-order lath shows crystal lattice fringes up to the edges of the grain (arrow); no amorphous phases (neither inorganic nor organic) are detected here. (*e*) Irregularly shaped aragonite grain (bright-field image) with polycyclic twinning lamellae; (*f*) dark-field image of the same grain (foil #2592).

We analysed six TEM foils cut from the same polished section for which EBSD measurements were performed (figure 1*a*). Prominent characteristics observed in the TEM analyses are the typical particulate nature, well described for natural biominerals (e.g. [29,32,33]) associated with multi-scale porosity throughout the shell (figures 4–6). The outer shell layer (figure 4) displays micrometre-sized cavities between the third-order laths (figure 4*a*,*b*), while the inner shell is less porous at the micrometre scale (figure 5*a,b*). By contrast, the inner shell shows submicrometre porosity *within* the third-order aragonite laths (figure 5*c,d*), which we found less frequently in the foils cut from the outer part of the shell. We observed numerous and variably sized, irregular voids (approx. 30–130 nm in size) rimming the third-order lath boundaries, with smaller voids (approx. 5–15 nm in size) *within* these aragonite laths (figure 5*c,d*, arrows). They appear to be focused along the outer areas of the laths in the inner shell layer, resulting in irregular 50–100 nm wide concentration zones of voids. While the foil cut perpendicular to the long axis of the aragonite laths (figure 5*c*) shows these voids apparently distributed at random, the rims of the laths cut parallel to their longest axes in figure 5*d* are not completely enclosed by a focus zone of voids. It is possible that this apparent preferential focus in figure 5*d* is a sectioning effect from foil preparation.

Crystal shapes in both layers of the shell are highly irregular (figure 6*a–f*) and aragonites commonly display variable and strongly ‘speckled’ diffraction contrasts in TEM bright and dark field imaging, where different areas within an individual grain display sharply different diffraction contrasts (e.g. figure 6*b*, arrow). Polycyclic/polysynthetic twinning is common at the nanoscale in both shell layers (figures 5*c* and 6*e,f*).

All areas studied by high resolution TEM were found to be crystalline, even the rim areas of the grains (figure 6*d*), which contrasts with nacroprismatic shells, where amorphous areas are observed [30,34], amounting to 10 at% in some shells [35]. Nevertheless, results from solid-state nuclear magnetic resonance spectroscopy on the same *T. derasa* shell sample indicate an overall amount of 3–7 at% of amorphous calcium carbonate [35], showing that while this phase is present in the shell, it was apparently not sampled by any of the TEM foils in this study.

### 4.3. Thermogravimetric analysis

**Figure 7.**
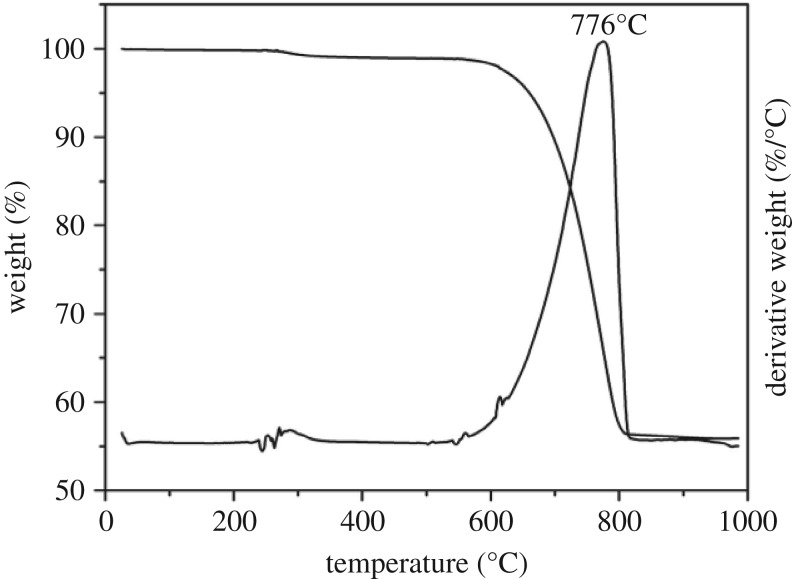
TGA/DTG analyses. The range 150–500°C was used for calculation of the total organic matrix content. The peak at 776°C marks the decomposition of calcium carbonate and release of CO_2_.

Thermogravimetry was used to quantify water and organic contents in a powdered sample of the shell. The total organic matrix amounts to 0.94 wt% as calculated from the integrated mass loss in the temperature range 150–500°C (figure 7). Peaks at around 300°C are attributed to the decomposition of organic macromolecules. Aragonite converts to calcite at approximately 500°C, as verified by FTIR spectrometry, and decomposition to CaO and CO_2_ is completed at around 800°C (figure 7).

### 4.4. Fourier transform infrared spectroscopic characterization of the organic matrix

**Figure 8.**
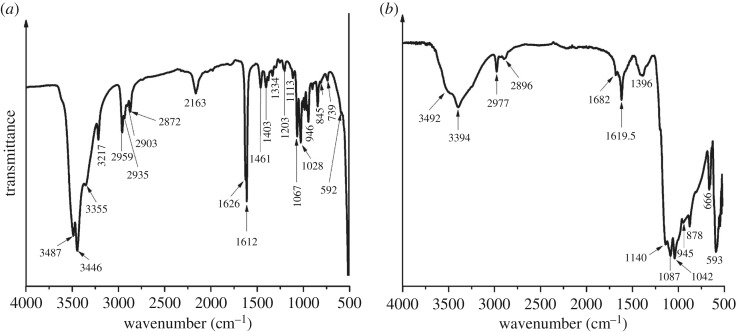
(*a*) FTIR spectrum of the total organic matrix in the range of 4000–500 cm^−1^. (*b*) FTIR spectrum of lipids and lipoproteins extracted from the shell sample. For band assignments see table 1.

FTIR analysis of the total organic matrix extract of the shell reveals prominent structural chitin-protein bands (table 1): bands indicative for amide A (3000–3500 cm^−1^), amide B (2800–2990 cm^−1^), amide I (1600–1700 cm^−1^), amide II (1300–1590 cm^−1^) and amide III (1190–1290 cm^−1^) are present. Strong broad bands between 3200 and 3450 cm^−1^ (figure 8*a*) are characteristic for OH and/or NH stretching modes. The band at 1403 cm^−1^ (figure 8*a*) is assigned to the C=O stretching of structural proteins and amino acids. Other major bands at 1113 cm^−1^, 1067 cm^−1^ and 1028 cm^−1^ represent sugars in the chitin structure [38,39] (table 1). The band at 1626 cm^−1^ corresponds to the C=O stretch in amide I and indicates β-sheet structure of the chitin [40]. The β-sheet structure was confirmed by measuring the optical activity of the soluble organic matrix in solution where the chitin was found to have a stable negative optical rotation of −25° after seven days [41].

**Table 1.** Band assignments for the main bands in the FTIR spectra (cm^−1^) of the shell organic matrix components for *T. derasa.* Band assignments carried out using data from [36–38].

total organic matrix	band assignment
3487, 3446	*v*_OH_
3355, 3217	*v*_N–H_
2959	*v*^as^_C–H of alkyl_
2935	*v*^s^_C–H of methylene_
2903, 2872	*v*^s^_C–H of alkyl_
1626, 1612	*v*_C=O_
1462	*δ*_C–H of alkyl_
1403	*v*^s^_C=O_
1334	*δ*_C–N_ + δ_N–H_
1379	*δ*_C–H_ + δ_C–CH_3__
1255	*δ*_N–H_
1203	*v*_C–O_
1298	*v*_C–O_
1028, 1067, 1113	*v*_C–O–H_
987	*γ*_C–H of alkyl_
962, 946	*γ*_C–H of methylene_
902, 845	*γ*_C–H_
739	*ρ*_C–H of methylene_
592	*γ*_C–C_

A well-defined band at 1461 cm^−1^ (figure 8*a*) is attributed to amide II (table 1), while bands at 1203 cm^−1^ and 1255 cm^−1^ are the carbonyl stretching and amide deformation vibrations of amide III (table 1). The OH and/or NH functional groups for amide A and the C–H stretching modes for amide B have many prominent bands in the soluble organic matrix, for example, at 3487, 2959 and 2872 cm^−1^.

Major bands between 1000 and 1150 cm^−1^ [42,43], and 952–980 cm^−1^ [39] are indicative of sugars. The carbonyl bands (C–O–H) between 1113 and 1028 cm^−1^, and C–H vibrational bands between 946 and 987 cm^−1^ (table 1) are most likely C–O stretching vibrations in *N*-acetylglucosamine in the sugars [39]. The bands at 845 and 902 cm^−1^ are characteristics of the β-configuration in the anomeric centre [44], confirming the presence of polysaccharides.

The extracted lipids and lipoproteins (figure 8*b*) show indicative bands at 3394 cm^−1^ (O–H), 1682 cm^−1^ (amide I) and 1619.5 cm^−1^ (C=C), while sugar bands are seen between 1150 and 1000 cm^−1^ and are strong in the fingerprint region at 666 and 593 cm^−1^.

## 5. Discussion

### 5.1. Characteristics of the multi-scale shell architecture and organic moiety

Compared with the prism-nacre microstructure of mollusc shells, the crossed-lamellar architecture of the *T. derasa* shell is highly mineralized with only 0.9 wt% total organic content compared with greater than 3 wt% in nacre [45]. Its structure is distinctly different and overall more complex than the nacro-prismatic structure [12], with three hierarchy orders of aragonite laths creating an interlocking fabric reminiscent of plywood (figure 1) [7,8]. Some authors have interpreted polycyclic aragonitic growth twins occurring in the third-order structure as a fourth hierarchical order (e.g. [15,46,47]).

In *T. derasa* shells, the first order lamellae run approximately perpendicular to the growth layers (GL in figure 2), and radially with respect to the shell surface. They comprise thin particulate lamellae with high aspect ratios (second- and third-order structures: figure 1*b*), showing voids along grain boundaries, and within grains at the nanoscale (figure 5*c,d*) that contain organic macromolecules [48]. In contrast to nacro-prismatic bivalve shells, whose architecture consists of well-defined inter-crystalline organic matrix (the so-called interlamellar membranes) interlayered with mineral incorporating intra-crystalline organic matrix molecules, the crossed-lamellar *T. derasa* shell does not show a similarly prominent organic inter-crystalline framework [49].

Within the first-order lamellae, the CPO map (figure 2*a*) shows areas of second-order lamellae that are highly co-oriented, meaning that crystal co-orientation of aragonites for these areas is coherent across the organic layers between the aragonite laths as well as the organic growth lines shown in figure 1*e*. Similar aragonite tablets highly co-aligned across their organic envelopes occur in nacre [50] and are thought to form by a combination of epitaxial crystallization via mineral bridges across the organic envelopes [51] as well as due to competition for space upon growth [52]. It is conceivable that a similar model could be proposed for the formation of the domainal organization in crossed-lamellar structures.

Voids are a characteristic feature of biominerals and are common in bivalve shells irrespective of their microstructure [3,48,53,54]. The voids shown by the TEM analysis (figures 4–6) have larger diameters along the grain rims, are smaller within the aragonites (figure 5*c*) and are more common and larger in the outer shell layer (figure 4) than in the innermost layer of the shell (figure 5). Voids in nacre platelets are smaller (2–40 nm) than found in this study [48,53,54]. They are focused towards the inner part of the nacre platelets and form an approximately 50 nm wide void-depleted zone along the outer platelet rim adjacent to the interlamellar organic sheets [48]. This contrasts with our observations, where small voids are more common along the outer areas of each grain (figure 5*c,d*).

While mollusc shell microstructures differ distinctly at the micrometre and millimetre scale, a common structural motif exists at the nanoscale. It is an almost universal characteristic of biominerals that they are granular at the nanoscale [35]. The granular features have sizes in the range of tens of nanometres, are embedded in an intergranular organic medium (or cortex), and are easily observed by phase-contrast atomic force microscopy (e.g. [13,32,55,56]), and their presence is supported using TEM (e.g. [29,30]): irregular grain boundaries (figures 5*c* and 6*b,e,f*) and small voids (figure 5*c,d*) outline the shapes of the nanogranules in the TEM images. This granular nanostructure in mollusc shells is a consequence of their colloid-mediated, non-classical mode of growth [57,58].

The organic shell matrix in *T. derasa* consists of a mixture of polysaccharides as well as glycosylated and unglycosylated proteins and lipids (figure 8*a*), which is a rather typical general organic assemblage in mollusc shells [1,59]. The polysaccharides play a major role providing organic ‘scaffolding’ for the mineralized part (e.g. [1,60]), but may also have an active function of lowering the energy barriers for mineral nucleation [61]. The presence of polysaccharides, not only in nacro-prismatic mollusc shells but also in shells with crossed-lamellar microstructure, reiterates the applicability of the general biomineralization models that promote polysaccharides as the major organic template in this process (e.g. [62]).

### 5.2. Aspects of mechanical properties of the crossed-lamellar microstructure

The strength and toughness of shells are specifically distinct and superior to non-biogenic aragonite [5], with the crossed-lamellar shell microstructure displaying the highest fracture toughness [3]. These emergent mechanical properties of shells are a consequence of a combination of parameters including (but not limited to) the complex, multi-order hierarchy [3], their nano-granular texture [63], the organic–inorganic nanocomposite nature of the material [64,65], and high flaw tolerance at the nanoscale [66].

Crystallographically, the crossed-lamellar structure of the *T. derasa* shell belongs to a family of similar, but not identical structures. In a comprehensive study of 40 mollusc species including a closely related species, *Tridacna gigas,* Almagro *et al*. [12] distinguished five crystallographic groups of crossed-lamellar structures. Among these, *Tridacna* shells display a strong fibre texture with highly oriented crystallographic *c*-axes, and randomly oriented *a* and *b* axes (figure 2*c*). In fact, the texture of *T. derasa* (figure 2*d* for scale of textural index) ranges among the strongest mollusc shell textures (e.g. [12,67,68]).

**Figure 9.**
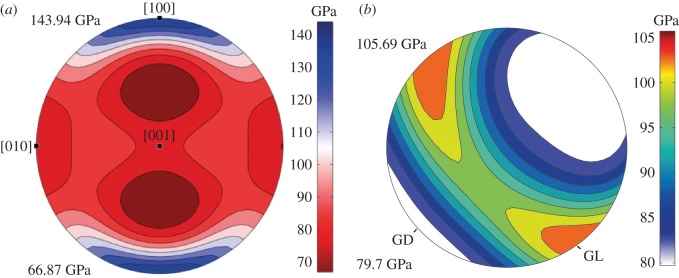
Young's modulus derived from the crystallographic preferred orientation for the *T. derasa* shell. (*a*) Young's modulus for a single crystal of aragonite calculated via the Voigt–Reuss–Hill averaging scheme based on the aragonite elastic constants by De Villiers [70]. (*b*) Young's modulus for the EBSD orientation map in figure 2*a* via Voigt–Reuss–Hill averaging scheme for the aggregate elastic constant (using the single crystal elastic constants of [70]). Note the development of a plane of isotropy following the distribution of aragonite [100] and [010] axes, leading to the optimization of general stiffness in all directions along the growth lines. Numbers in GPa are Young's modulus maximum and minimum values (cf. scale).

The aragonite crystallographic *c*-axes ([001]) in the *T. derasa* shell are oriented perpendicular to GL (figure 2*c*), thus, are radially oriented with respect to the shell surface. Aragonite single crystals are elastically anisotropic [69], with the anisotropy of the Young's modulus reaching values around 50% between the weakest and stiffest axes (figure 9*a*). The stiffest axis is [100] with a Young's modulus value of approximately 140 GPa, whereas the [010] and [001] axes are considerably weaker with values of around 80 GPa (figure 9*a*). The weakest direction in an aragonite single crystal lies at an intermediate position between [100] and [001]. By averaging the elastic constant of the *T. derasa* shell based on its crystallographic preferred orientation, it is possible to estimate the general stiffness of the shell based on the elastic properties of the aragonite single crystals. The CPO shows that [100] and [010] are orientated along a girdle around [001]. Such a CPO leads to the development of a plane of quasi-isotropy of the Young's modulus parallel to the girdle of the stiff axis [100] and the weak axis [010] (figure 9*b*). This minimizes the natural elastic anisotropy of aragonite in this plane while optimizing the general stiffness and resistance of the shell in all directions. Ouhenia *et al*. [71] recognized a similar strategy for shells of the gastropod *Charonia lampas lampas*: these shells consist of a stack of three different crossed-lamellar layers, whose alternated orientations maximize the stiffness coefficients in all directions of the whole shell.

This mechanical effect is further enhanced by the crystal orientation angle spread due to twinning. The combined use of TEM and EBSD shows that twinning, which is common at the nanoscale, remains a prominent structural motif across all hierarchical orders with at least 26% twin boundaries (as detected at the millimetre scale across the CPO map by EBSD; figure 2*b*).

Most likely, this complex architectural design is aimed at increasing isotropy [6], adding to other strategies aimed at optimizing mechanical properties and providing a significant evolutionary advantage by generating higher rigidity and wear resistance that is beneficial to the organism's protection.

## 6. Conclusion

*Tridacna derasa* shells are highly mineralized bio-ceramics consisting of aragonite with around 1 wt% organics, namely polysaccharides, glycosylated and unglycosylated proteins and lipids. The shells have a crossed-lamellar microstructure with a strong fibre texture in which the aragonite crystallographic *c*-axes are aligned radially to the shell surface. The spread of crystal orientation angles due to intense twinning at all hierarchical structures, nano-granularity and random orientation of the aragonitic crystallographic *a* and *b* axes, optimize the Young's modulus of the shell in all directions and all spatial scales, thus increasing isotropy. This is one of the first comprehensive studies that identify optimization strategies for mechanical properties across all hierarchal structures in the shell.

## Data accessibility

All data are included in this article.
